# Effect of Fe_3_O_4_ and CuO Nanoparticles on Morphology, Genotoxicity, and miRNA Expression on Different Barley (*Hordeum vulgare* L.) Genotypes

**DOI:** 10.1155/2021/6644689

**Published:** 2021-02-03

**Authors:** Anastasija Petrova, Ilona Plaksenkova, Inese Kokina, Marija Jermaļonoka

**Affiliations:** Institute of Life Sciences and Technology, Department of Biotechnology, Daugavpils University, Daugavpils LV-5401, Latvia

## Abstract

Metal nanoparticles (NPs) have an influence on plant growth and development. They can alter plant shoot and root length, fresh biomass production, and even influence the genome. Nanoparticles are also able to affect expression levels of plant microRNAs. MicroRNAs are able to protect plants from biotic stress, including pathogens which cause powdery mildew. In this study, *Hordeum vulgare* L. varieties “Marthe” and “KWS Olof” were grown in hydroponics with magnetic iron oxide (Fe_3_O_4_) and copper oxide (CuO) NPs added at 17, 35, and 70 mg/L. Plant morphology, genotoxicity, and expression of miR156a were investigated. The Fe_3_O_4_ and CuO NPs demonstrated different effects on the barley varieties, namely, Fe_3_O_4_ nanoparticles increased plant shoot and root lengths and fresh biomass, while CuO nanoparticles decreased them. CuO NPs presence caused larger changes on barley genome compared to Fe_3_O_4_ NPs. Thus, Fe_3_O_4_ NPs reduced genome stability to 72% in the “Marthe” variety and to 76.34% in the “KWS Olof” variety, while CuO NPs reduced genome stability to 53.33% in “Marthe” variety and in the “KWS Olof” variety to 68.81%. The miR156a expression levels after Fe_3_O_4_ NPs treatment did not change in the “Marthe” variety, but increased in the “KWS Olof” variety, while CuO NPs treatment increased miRNA expression levels in the “Marthe” variety but decrease them in the “KWS Olof” variety. As NPs are able to influence miRNA expression and miRNAs can affect the plant resistance, obtained results suggest that tested NPs may alter plant resistance response to pathogens.

## 1. Introduction

Nanotechnology and its diverse products are an integral part of the modern lifestyle. Nanoparticles (NPs) are extensively used in agriculture in many cases including microfertilizers, pathogen detection, and pest control. Nanoparticles are also used in cosmetic products such as deodorants, as well as in household care products. Nanoparticles are also widely used in biomedicine as drug carriers [1, 2]. The use of NPs offers higher efficiency compared to larger particles, but NPs have unique functional capabilities, electrical and optical properties, high stability, and high adsorption capacity [3]. Nanoparticles have a major influence on plant morphology and genome. A small amount of NPs can increase crop yields, but too much NP exposure can cause physiological disturbances in plants as well as oxidative stress. In addition, NPs are able to reduce the activity of antioxidative enzymes leading to cytotoxicity and genotoxicity [4, 5].

Iron is one of the most important plant nutrients for plant development, but copper is a micronutrient which assists in plant metabolism. Fe_3_O_4_ and CuO NPs are used in small doses as fertilizers for soil to enrich the essential metal content, thus augmenting crop growth. Iron oxide and copper oxide NPs are used in the form of fungicides in high doses to protect plants against pathogens [6–8]. Plants that had CuO NPs applied at 150–340 *μ*g/mL had superior fungal treatment results compared to those treated with Cu_2_O and Cu/Cu_2_O NPs [9]. Application of 50 mg/L CuO NP suspension to rose leaves reduces the growth of *Podosphaera pannosa* fungi [10]. Fe_3_O_4_ NPs can increase *Nicotiana benthamiana* plant resistance response against the tobacco mosaic virus, developing plant morphological parameters such as plant dry and fresh weights [11]. Metal-based NPs have a major impact on plant morphology. Fe_3_O_4_ NPs are able to increase the length of tomato, wheat, and lettuce roots. CuO NPs' different concentrations can reduce shoot and root length in chickpea plants. Seed germination of cucumber, rice, lettuce, and radish was reduced under CuO NPs stress [5, 12, 13].

Plants have broad-spectrum defense mechanisms against pathogens [14]. Vertical (specific) and horizontal (nonspecific) resistances to plant diseases play a general role in plant protection across infections. Nonspecific resistance protects plant varieties against multiple pathogens, which is encoded by the recessive mutation allele (mlo) of the *MLO* gene. The *MLO* gene is a negative regulator of cell death and a regulator of powdery mildew [15, 16]. In barley, powdery mildew fungi are able to penetrate the host cell wall by using *MLO* proteins, leading to spontaneous stem cell death. However, homozygous mutant recessive (*mlo*) alleles of the *MLO* genes confer broad-spectrum disease resistance. Plants often show sensitivity, where the *MLO* genes generally express *MLO*'s respective proteins, which mediate cell-cell communication in plants. Importantly, there is broad-spectrum disease resistance when the *MLO* gene is not expressed or when it expresses dysfunctional proteins [17–22].

MicroRNA (miRNA) is an endogenous small noncoding RNA, which consists of 18–24 nucleotides. miRNA plays a crucial role in the regulatory functions responsible for gene expression in eukaryotic organisms—miRNA complementarily binds to the target messenger RNA (mRNA) and facilitates its degradation, thus suppressing gene expression [23–25]. Plant miRNA not only regulates various aspects of plant development but has been implicated in resistance to biotic stresses as well as in regulating immunity to pathogens (viruses, bacteria, and fungi) [26]. The miR156 is miRNA which has a regulatory role in plants and responds to biotic stress, hypoxia, salt stress, and stress induced by NPs. The miR156 family is associated with plant response to the pathogens and is able to control bacterial, fungal, and viral diseases in plants [24, 27–29].

Unstable weather conditions, especially the humidity developing in agricultural regions, contribute to the spread of pathogenic diseases in plants, such as powdery mildew, which immensely reduces cereal yields and consequentially affects global markets [30]. To avoid pathogenic diseases, the agricultural industry uses different fungicides or potassium fertilizers to reduce plant exposure to fungal infections. Also used is a mix of cultivated crop varieties, which are partially or completely susceptible to pathogens [31, 32].

As an example, barley (*Hordeum vulgare* L.) is a one-year cereal plant which is widely used not only in agriculture, forage, and malt but also in the food industry worldwide [33–36]. Barley, like other cereals, is affected by various diseases, most often caused by pathogens. The most persistent control against pathogens is the use of resistant barley varieties (for example, varieties with different *MLO* genes). Using pathogen-resistant varieties automatically increases yield in their growing regions [30].

The aim of this study was to investigate the effects of different concentrations of Fe_3_O_4_ and CuO NPs on seedling morphology, genotoxicity, and *mlo*-resistance-related miRNA expression on different barley varieties.

## 2. Materials and Methods

### 2.1. Nanoparticles Characteristics

Samples of 25 nm of Fe_3_O_4_ NPs and 25 nm of CuO NPs were provided by G. Libert's Center of Innovative Microcopy, Daugavpils University. Nanoparticles were diluted in G. Libert's Center in water to 17 mg/L, 35 mg/L, and 70 mg/L and sonicated for 1 h to split the formed nanoparticle agglomerates into individual NPs.

### 2.2. Plant Materials and Growth Conditions

Seeds of two varieties of *Hordeum vulgare* L. “Marthe” and “KWS Olof” were provided by the Institute Of Agricultural Resources and Economics, Stende Research Center (Priekuli, Latvia). The “Marthe” variety has a *mlo*11 gene, and the “KWS Olof” variety has a *mlo* (unknown) gene. The seeds were rinsed with deionized water and transferred to Petri dishes and kept in plates at 22°C for 1 day. The seeds were transferred to a hydroponic solution with 50% Murashige and Skoog (MS) salt solution and kept in a climate chamber 16 h/8 h day/night photoperiod at 23°C [37]. One-week-old seedlings were divided into six experimental groups and transferred to an aqueous hydroponic solution with NPs: three experimental groups were plants grown in 3 mL of different concentrations of Fe_3_O_4_ NPs (17 mg/L, 35 mg/L, and 70 mg/L), three experimental groups were plants grown in 3 mL of different concentrations of CuO NPs (17 mg/L, 35 mg/L, and 70 mg/L), and control group plants were grown in water. The plants were watered daily with tap water and fertilized with fresh 50% MS salt solution every day. Two-week-old fresh barley seedlings were taken for plant morphology analysis, genotoxicity detection, and miRNA level determination.

### 2.3. Measurement of Seedling Biomass, Shoot, and Root Length

The morphological parameters of the control and experimental barley (*H. vulgare* L.) varieties “Marthe” and “KWS Olof” were measured by length of shoot, length of root, and fresh plant biomass using the method followed by [37].

### 2.4. DNA Extraction and Randomly Amplified Polymorphic Analysis

The genotoxic effects induced by Fe_3_O_4_ and CuO NPs were assessed using the randomly polymorphic DNA (RAPD) technique. Genomic DNA was extracted from 140 samples from each variety of fresh barley shoots (20 in each experimental group). Extraction was carried out via GeneMATRIX Swab-Extract DNA Purification Kit following the purification of total DNA protocol. The quantity and quality of genomic DNA was assessed using a spectrophotometer (NanoDrop One, Thermo Scientific, USA).

A total of three decamer primers, OPA-05 (5′-AGGGGTCTTG-3′), OPA-07 (5′-GAAACGGGTG-3′), and OPD-18 (5′-GAGAGCCAAC-3′), were selected for the RAPD analysis. The RAPD PCR program started with initial denaturation at +94°C for 1 min, followed by 35-cycle consisting of a denaturation step at +94°C for 1 min, an annealing step at +35°C for 90 s, an extension of product at 72°C for 2 min per 1 kb, and the final extension set at +72°C for 10 min using the Thermal Cycler UNO96 (*VWR*, United Kingdom). The PCR reaction products were checked with QIAxcel Advanced (Qiagen, Germany) capillary electrophoresis instrument according to [28] and utilizing a tiTaq PCR Master Mix (2x) kit (Poland, Gdańsk) according to the protocol fixation of DNA fragment sizes using with QIAxcel ScreenGel Software (Qiagen, Germany).

### 2.5. Detection of Genotoxicity by Estimation of Genomic Template Stability

The Genome template stability (GTS) value is calculated for each primer using the formula reported by Rocco et al. [38]:

(1) $$\begin{array}{c} \mathrm{GTS\%}=\left(\frac{1-a}{n}\right)\times 100, \end{array}$$

where *a* is the average number of polymorphic bands detected in each sample, and *n* is the total number of bands in the control samples. The polymorphism in RAPD profiles involves the disappearance of the normal band and the appearance of a new band relative to control. To compare the sensitivity of each parameter, changes in these values are calculated as percentages [38, 39]. Genome template stability values were calculated for each experimental group.

### 2.6. Expression Validation of MicroRNA Using Real-Time qPCR

Two-step quantitative real-time PCR (qRT-PCR) analysis was performed to assess miRNA expression levels in barley varieties “Marthe” and “KWS Olof” grown with different concentrations of Fe_3_O_4_ and CuO NPs compared to control plants. miRNAs were extracted from the shoots using a Universal RNA/miRNA Purification Kit (EURx, Poland). RNA was extracted from 140 samples from “Marthe” and “KWS Olof” variety fresh shoots (20 in each experimental group). RNA was quantified and qualified with a spectrophotometer (NanoDrop One, Thermo Scientific, USA). Samples with an A260/280 ratio from 1.7 to 2.1 were used for analysis. miRNA target-specific primer hvu-miR156a with locked nucleic acid was designed. The target miRNA hvu-miR156a sequence was 5′-TGACAGAAGAGAGTGAGCACA-3′. HvsnoR14 was used as a reference gene for the normalization of miRNA expression values. Reverse transcription for miRNAs was performed using the Thermal Cycler UNO96 (VWR, United Kingdom) and miRCURY LNA RT Kit (Qiagen, Germany) according to the protocol first-strand cDNA synthesis. For miRNA qRT-PCR analysis, miRCURY SYBR Green PCR kit (Qiagen, Germany) was used according the manufacturer's protocol. The Rotor Gene Q Series Software program was used to analyse the miR156a expression level of barley varieties. The results were analysed using the 2 − ΔΔCT method [40].

### 2.7. Statistical Analysis

The results were expressed as an average for the measurement and were reported with ±SD. Student's *t*-test was used to determine statistical differences and significant means of the experimental data examination. In all statistical analyses, the significant differences were determined at a *p* value of 0.05 or 0.01. All of the experimental values were compared to their relevant control. All of the experiments were repeated three times.

## 3. Results and Discussion

### 3.1. Plant Morphology

Differences in shoot and root length and in fresh plant biomass were found for each barley variety compared to their respective controls, where different Fe_3_O_4_ and CuO NP concentrations of 17 mg/L, 35 mg/L, and 70 mg/L were used.

**Figure 1:**
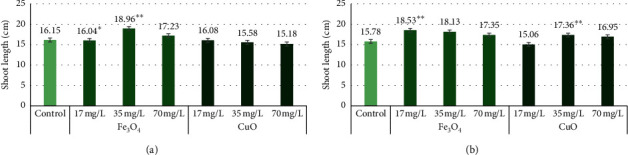
The “Marthe” and “KWS Olof” varieties' shoot length with 17 mg/L, 35 mg/L, and 70 mg/L concentrations of Fe_3_O_4_ and CuO NPs. Values are the mean of three replicates with SD. ^*∗*^Indicates a significant difference from control (*p* < 0.05), and ^*∗∗*^indicates a significant difference from control (*p* < 0.01). (a) “Marthe” variety shoot length with different concentrations of Fe_3_O_4_ and CuO NPs. (b) “KWS Olof” variety shoot length with different concentrations of Fe_3_O_4_ and CuO NPs.

Comparing the effect on shoot length among the different concentrations of Fe_3_O_4_ and CuO NPs on “Marthe” and “KWS Olof” barley varieties, the 17 mg/L Fe_3_O_4_ NP treatment induced significant increase in the “Marthe” (*p* < 0.05) and “KWS Olof” (*p* < 0.01) varieties. The 35 mg/L Fe_3_O_4_ treatment also significantly increased shoot length only in the “Marthe” variety. CuO NPs at 35 mg/L significantly increased the shoot length of the “KWS Olof” variety only (*p* < 0.01). The “Marthe” variety control group shoot length was 16.15 cm, and the groups with Fe_3_O_4_ NPs at 17 mg/L shoot length was 16.04 cm, the 35 mg/L group was 18.96 cm, and the 70 mg/L group was 17.23 cm. The same variety with CuO NP treatment at 17 mg/L was 16.08 cm, 35 mg/L was 15.58 cm, and 70 mg/L was 15.18 cm (Figure 1). The “KWS Olof” variety control group shoot length was 15.78 cm, and the groups with Fe_3_O_4_ NP treatment at 17 mg/L shoot length was 18.53 cm, 35 mg/L was 18.13 cm, and 70 mg/L was 17.35 cm. The same variety with CuO NP treatment as 17 mg/L achieved 15.06 cm, 35 mg/L was 17.36 cm, and the 70 mg/L group was 16.95 cm (Figure 1). Only the Fe_3_O_4_ NP treatment at 17 mg/L in the “Marthe” variety decreased shoot length compared to the control. All other Fe_3_O_4_ NPs concentrations, including 17 mg/L concentration, increased the shoot length of both barley varieties. CuO NPs at all concentrations decreased the “Marthe” variety shoot length, but in the “KWS Olof” variety, only CuO NPs at the 17 mg/L concentration decreased shoot length; all other concentrations of CuO NPs in this variety increased shoot length.

**Figure 2:**
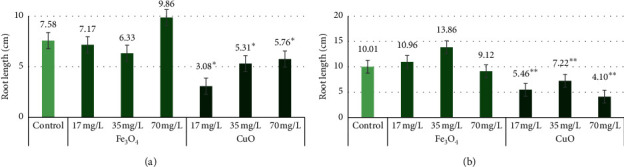
The “Marthe” and “KWS Olof” varieties root length with 17 mg/L, 35 mg/L, and 70 mg/L concentrations of Fe_3_O_4_ and CuO NPs. Values are the mean of three replicates with SD. ^*∗*^Indicates a significant difference from control (*p* < 0.01). (a) “Marthe” variety root length with different concentrations of Fe_3_O_4_ and CuO NPs. (b) “KWS Olof” variety root length with different concentrations of Fe_3_O_4_ and CuO NPs.

Different Fe_3_O_4_ NP concentrations insignificantly affected “Marthe” and “KWS Olof” varieties' root length. All CuO NP concentrations significantly (*p* < 0.05) decreased “Marthe” root length, and all CuO NP concentrations significantly (*p* < 0.01) decreased “KWS Olof” root length. The “Marthe” control group root length was 7.58 cm, and the group with Fe_3_O_4_ NPs at 17 mg/L root length was 7.17 cm, 35 mg/L was 6.33 cm, and 70 mg/L was 9.86 cm. The “Marthe” group with CuO NPs at 17 mg/L was 3.08 cm, 35 mg/L was 5.31 cm, and 70 mg/L was 5.76 cm (Figure 2). Among the “KWS Olof” variety, the control group root length was 10.01 cm, and the group with Fe_3_O_4_ NPs at 17 mg/L root length was 10.96 cm, 35 mg/L was 13.86 cm, and 70 mg/L was 9.12 cm. The “KWS Olof” group with CuO NPs at 17 mg/L was 5.46 cm, 35 mg/L was 7.22 cm, and 70 mg/L was 4.1 cm (Figure 2). Fe_3_O_4_ NPs at 17 mg/L and 35 mg/L decreased “Marthe” variety root length and 70 mg/L concentration decreased “KWS Olof” variety root length. In all other cases, Fe_3_O_4_ NPs increased root length.

**Figure 3:**
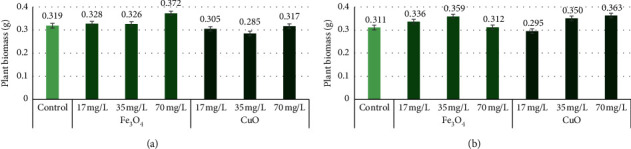
The “Marthe” and “KWS Olof” varieties fresh plant biomass with 17 mg/L, 35 mg/L, and 70 mg/L concentrations of Fe_3_O_4_ and CuO NPs. Values are the mean of three replicates with SD. (a) “Marthe” variety fresh plant biomass with different concentrations of Fe_3_O_4_ and CuO NPs. (b) “KWS Olof” variety fresh plant biomass with different concentrations of Fe_3_O_4_ and CuO NPs.

All Fe_3_O_4_ NP concentrations had a positive effect on “Marthe” and “KWS Olof” varieties' plant fresh biomass—in each experimental group, biomass increased compared to the control plant. However, Fe_3_O_4_ and CuO NPs at 17 mg/L, 35 mg/L, and 70 mg/L concentrations did not significantly affect all varieties seedling fresh biomass. CuO NPs at all concentrations decreased “Marthe” variety plant biomass. CuO NPs at 17 mg/L concentration decreased fresh plant biomass, but 35 mg/L and 70 mg/L concentrations increased in the “KWS Olof” seedling biomass. The “Marthe” variety control group fresh plant biomass was 0.319 g, and the group with Fe_3_O_4_ NPs at 17 mg/L plant biomass was 0.328 g, 35 mg/L was 0.326 g, and 70 mg/L was 0.372 g. For CuO NPs, at 17 mg/L, plant biomass was 0.305 g, 35 mg/L was 0.285 g, and 70 mg/L was 0.317 g (Figure 3). The “KWS Olof” variety control group fresh plant biomass was 0.311 g, and the group with 17 mg/L Fe_3_O_4_ NPs was 0.336 g, 35 mg/L was 0.359 g, and 70 mg/L was 0.312 g. With CuO NPs, at 17 mg/L, fresh biomass was 0.295 g, 35 mg/L was 0.3504 g, and 70 mg/L was 0.363 g (Figure 3).

It is known that the degree of metal bioaccumulation from CuO NPs may be affected by the NP evaporation rate [7]. In general, NPs based on metals such as CuO can dissolve and release metal ions [41]. In the same way, the CuO NPs accumulation in plants depends on the concentration of NPs—bioaccumulation has been shown to increase with increasing CuO NPs concentration in wheat, mung bean, zucchini, and lettuce [42, 43]. As a result, experimental plants with CuO at different NP concentrations do not exhibit an exponential decrease of *H. vulgare* L. root length. Also, Shawn et al. [44] showed that CuO NPs at different concentrations (0.5 mM, 1.0 mM, and 1.5 mM) regularly decrease *H. vulgare* L. root length and shoot length with increasing NP concentrations compared to control. Similarly, Zakharova et al. [45] presented results where CuO NPs at 0.01 g/L, 0.1 g/L, and 1 g/L reduced *Triticum aestivum* L. root length. Also, Wrigth et al. [46] observed the same trend in wheat but with different CuO NP concentrations. The AlQuraidi et al. [47] study showed results where CuO NPs concentrations (200, 400, and 800 mg/L) decreased plant fresh biomass and root length in coriander (*Coriandrum sativum*). Moreover, Margenot et al. [48] showed that 16 nm CuO NP concentrations affected carrot (*Daucus carota* subsp. sativus cv. Little Finger) and lettuce (*Lactuca sativa*, cv. Nevada Summer Crisp) root thickness, reducing it. The Wang et al. [49] study results showed that CuO NPs doses (50 and 500 mg/kg) applied to *T. aestivum* inhibited plant growth, reducing plant biomass and shoot lengths. A study with peanut (*Arachis hypogaea* L.) showed that CuO NPs 50 mg kg^−1^ concentration inhibited plant growth, as well as reduced plant biomass and shoot length [50].

In the present study, it was shown that Fe_3_O_4_ NPs at 17 mg/L, 35 mg/L, and 70 mg/L increased the shoot length, root length, and fresh seedling biomass of barley varieties. Tombuloglu et al. [51] presented results showing that increasing Fe_3_O_4_ NP concentrations (125 mg/L, 250 mg/L, 500 mg/L, and 1000 mg/L) irregularly increased plant shoot length, root length, and fresh *H. vulgare* L. plant biomass. Konate et al. [12] showed that Fe_3_O_4_ NPs at 2000 mg/L increases *Triticum aestivum* L. root length compared to control by a factor of 1.1, and that wheat shoot length was doubled compared to control. Moreover, the results showed that small concentrations of Fe_3_O_4_ NPs (5 mg/L, 10 mg/L, 15 mg/L, and 20 mg/L) also increased wheat plant root length [52]. Relatively low concentrations of NPs were used in this study with barley varieties compared to other studies, where most often the effect of highly concentrated NPs are studied.

### 3.2. Genotoxicity Analysis by RAPD Assay

The genotoxicity of Fe_3_O_4_ and CuO NPs was investigated by observing the band profile after the RAPD assay on 5 replicates per treatment obtained from different barley variety seedlings. All primers created stable RAPD bands. The genomic changes were noted as appeared (a) and disappeared (b) bands in treated plant DNA compared to control bands (Table 1). Changes in DNA bands (fragment dropouts or new fragment formations) in samples treated with NPs reflect DNA changes in the genome from single base changes (point mutations) to complex chromosome rearrangements considered to be genotoxic [38, 53].

**Figure 4:**
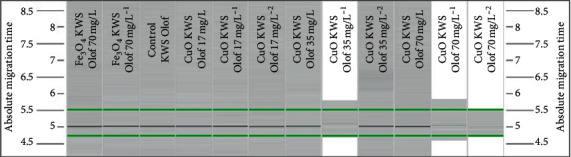
Examples of the RAPD profile of genomic DNA isolated from barley “KWS Olof” variety treated with Fe_3_O_4_ and CuO NPs.

The number of total bands varied from 6 (OPA-05 primer) to 30 (OPD-18 primer). The largest number of polymorphic bands (*n* = 14) showed when using primer OPA-05 analysing the “Marthe” variety treated with 17 mg/L of CuO NPs. The lowest number of polymorphic bands (*n* = 0) showed using primer OPA-05 to analyse the “Marthe” variety treated with 35 mg/L and 70 mg/L of Fe_3_O_4_ NPs and 70 mg/L with CuO NPs, as well as the “KWS Olof” treated with 17 mg/L of CuO NPs. Overall, 63 new bands appeared in treated plants with Fe_3_O_4_ and CuO NPs at 17 mg/L, 50 new bands appeared in treated plants with Fe_3_O_4_ and CuO NPs at 35 mg/L, and 59 new bands appeared in treated plants with Fe_3_O_4_ and CuO NPs at of 70 mg/L. However, 30 (17 mg/L), 28 (35 mg/L), and 29 (70 mg/L) disappeared bands were detected and compared with the control samples. The example of electropherogram is presented in Figure 4.

Genome template stability was calculated for all experimental plants. The genome stability for control plants was determined to be 100%.

Fe_3_O_4_ NPs at 17 mg/L, 35 mg/L, and 70 mg/L did not significantly affect the genotoxicity of barley varieties. Only the CuO NP treatment at 17 mg/L significantly (*p* < 0.01) decreased the “Marthe” variety's genome stability.

**Figure 5:**
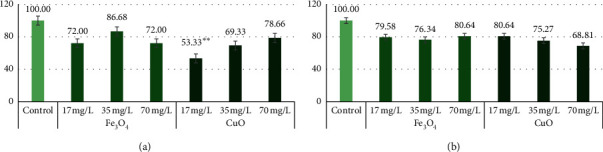
Genome template stability in “Marthe” and “KWS Olof” varieties exposed to 17 mg/L, 35 mg/L, and 70 mg/L Fe_3_O_4_ and CuO NPs concentrations. Values are the mean of three replicates with SD. ^*∗∗*^Indicates a significant difference from control (*p* < 0.01). (a) “Marthe” variety genome template stability (%) with Fe_3_O_4_ and CuO NPs. (b) “KWS Olof” variety genome template stability (%) with Fe_3_O_4_ and CuO NPs.

In “Marthe” variety plants treated with 17 mg/L and 70 mg/L Fe_3_O_4_ NPs, genome stability decreased to 72% and the 35 mg/L decreased to 86.68%. CuO NPs irregularly decreased genome stability. As the concentration of CuO NPs increased from 17 mg/L to 70 mg/L, the genome stability of the “Marthe” variety increased from 53.33% to 78.66%, respectively. In the “KWS Olof” variety, Fe_3_O_4_ NPs at 17 mg/L decreased genome stability to 79.58%, 35 mg/L to 76.34%, and 70 mg/L to 80.64% (Figure 5). CuO NPs decreased the stability of the genome from 80.64% (17 mg/L) to 68.81% (70 mg/L) as the concentration of nanoparticles increases (Figure 5). Analysing the two different impacts of NPs on the two barley variety's genomes, higher genotoxicity was caused by different CuO NP concentrations.

Compared to the study with *Medicago falcata* with 1 mg/L, 2 mg/L, and 4 mg/L concentrations of Fe_3_O_4_ NPs, the GTS increased as the concentration of NPs increased from 86.7% (1 mg/L) to 87.5% (4 mg/L) [37]. The same study with *Eruca sativa* plants with the same Fe_3_O_4_ NPs concentrations caused a GTS decrease as NP concentrations increased from 93.9% (1 mg/L) to 87.9% (4 mg/L) [28]. However, Tombuloglu et al. [51] showed that Fe_3_O_4_ NPs at 125 mg/L, 250 mg/L, 500 mg/L, and 1000 mg/L concentrations did not show any toxic effect on the experimental *H. vulgare* L. plants.

As result, the study with Cu NPs at 200 mg/L, 400 mg/L, and 800 mg/L shows that NP made significant changes in *C. sativum* plant genome using the RAPD technique. Primers OPA-01, OPA-02, and OPA-06 at all Cu NP concentrations showed the disappearance of one band for the genome, but primer OPA-07 has previously shown the formation of a new band at 400 mg/L and 800 mg/L NP concentrations [47]. The other study, which used Cu NPs at 50 mg/L, 100 mg/L, and 200 mg/L concentrations, showed a change to the *C. sativus* plant genome, where primers OPA-07 and OPA-08 formed new bands in the presence of NP [54]. In contrast, the different concentrations of nano-TiO_2_ and NaCl + nano-TiO_2_ applied to maize (*Zea mays* L.) affected GTS. The GTS values ranged from 28.8% to 87.7% [55]. The present study shows that CuO NPs at 17 mg/L, 35 mg/L, and 70 mg/L significantly and insignificantly decreased genome template stability in all experimental *H. vulgare* L. plants. The effect of CuO NPs on the plant genome and genotoxicity has not been studied previously.

### 3.3. MicroRNA Analysis

Referring to Gurjar et al. [56], miRNA expression levels are measured by the logarithmic formula Log_2_ (treatment/control). Each sample group with Fe_3_O_4_ and CuO NPs had different results.

Comparing the two barley varieties, treated with different Fe_3_O_4_ NPs concentrations, only 35 mg/L and 70 mg/L concentrations significantly (*p* < 0.01) increased the miR156a expression level in the “KWS Olof” variety. All concentrations of CuO NPs significantly (*p* < 0.01) increased miR156a expression levels in all barley varieties.

**Figure 6:**
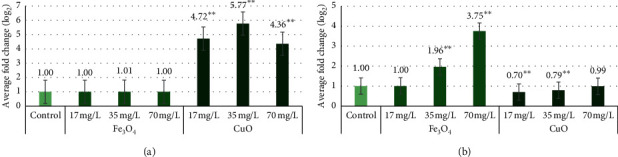
Average fold changes of the miR156a expression level in “Marthe” and “KWS Olof” variety grown with different concentrations of Fe_3_O_4_ and CuO NPs. Values are the mean of three replicates with SD. ^*∗∗*^Indicates a significant difference from control (*p* < 0.01). (a) miR156a expression level in “Marthe” variety with Fe_3_O_4_ and CuO NPs. (b) miR156a expression level in “KWS Olof” variety with Fe_3_O_4_ and CuO NPs.

All concentrations of Fe_3_O_4_ did not affect “Marthe” miRNA expression levels. miRNA expression levels remained at a 1.00-fold change with 17 mg/L and 70 mg/L concentrations and with 35 mg/L a 1.01-fold change. CuO NPs in the “Marthe” variety caused a strong increase in miRNAs expression levels—17 mg/L had a 4.72-fold change, 35 mg/L had a 5.77-fold change, and 70 mg/L had a 4.36-fold change (Figure 6). For “KWS Olof” variety, Fe_3_O_4_ NPs increased miR156a expression levels with increasing concentrations of NPs: 1.00-fold (17 mg/L), 1.96-fold (35 mg/L), and 3.75-fold (70 mg/L). In the “KWS Olof” variety, as the concentration of CuO increases, the miR156a expression level increases from 0.7-fold (17 mg/L) to 0.99-fold (70 mg/L) (Figure 6). Summarizing the data on the effect of Fe_3_O_4_ and CuO NPs, it can be concluded that, in this study, the expression level of miR156a was most increased in the “Marthe” variety by different concentrations of CuO NPs.

**Figure 7:**
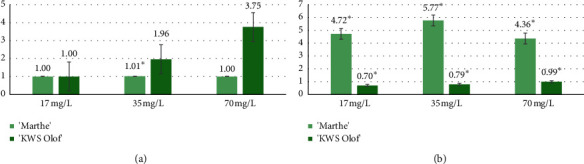
Comparison of 17 mg/L, 35 mg/L, and 70 mg/L concentrations of Fe_3_O_4_ and CuO NPs effects on the miR156a expression level in “Marthe” and “KWS Olof” varieties. Values are the mean of three replicates with SD. ^*∗*^Indicates a significant difference from control (*p* < 0.05). (a) Comparison of the different concentration Fe_3_O_4_ NP effect on miRNA expression in “Marthe” and “KWS Olof” varieties. (b) Comparison of the different concentration CuO NP effect on miRNA expression in “Marthe” and “KWS Olof” varieties.

For “Marthe” and “KWS Olof” varieties treated with Fe_3_O_4_ NPs at different concentrations, 35 mg/L treatment for all barley varieties give a statistically significant result comparing the varieties with each other, and 70 mg/L treatment comparing two barley varieties with each other, only the “KWS Olof” variety gives statistically significant data. Fe_3_O_4_ NP at 17 mg/L did not affect any barley variety's miRNA expression level, 35 mg/L and 70 mg/L concentrations did not affect “Marthe” miRNA level but increased the “KWS Olof” miR156 expression level. In case different concentrations of Fe_3_O_4_ NPs were applied to two *mlo*-barley varieties, in “KWS Olof” variety, Fe_3_O_4_ NPs at 35 mg/L and 70 mg/L gave a positive effect by increasing the miRNA expression level, resulting in increased plant resistance (Figure 7).

Barley varieties treated with different CuO NPs concentrations of 17 mg/L, 35 mg/L, and 70 mg/L show a vivid contrast between miRNA expression levels. All CuO NP concentrations have a statistically significant result comparing both varieties in all concentrations. CuO NP increased the “Marthe” variety miRNA expression level, but all copper oxide NP concentrations decreased the “KWS Olof” variety miRNA expression level. Different CuO NP concentrations increased only the “Marthe” variety miR156a expression level, increasing their resistance. The “KWS Olof” variety treated with CuO NPs shows only a negative effect on the miRNA expression level and decreased plant resistance (Figure 7).

In crop plants such as wheat, powdery mildew infection decreased miR156 expression levels [57]. Su et al. [21] suggested that nov-mir-10 reduced the expression level in sugarcane which can reduce the inhibition of defense response by the *MLO* protein and improve plant resistance to smut pathogen. Kokina et al. [37] studied *Medicago falcata* L. plants and showed that Fe_3_O_4_ NPs at 1 mg/L, 2 mg/L, and 4 mg/L increased the miR159c expression level at increased NPs concentrations by 0.31-fold, 0.36-fold, and 0.40-fold, respectively. The same study with *Eruca sativa* with the same Fe_3_O_4_ NP concentrations showed decreased miR159c expression levels with increased NPs concentrations by 1.30-fold, 1.19-fold, and 1.04-fold, respectively [28]. A study with TiO_2_ NPs showed that miR156 in *Nicotiana tabacum* is upregulated by 0.1% TiO_2_ but inhibited by 0.1% nano-TiO_2_ [58]. In contrast, 0.1% aluminium oxide NPs upregulated miR156 expression on tobacco plant with an insignificant fold change, but miR159 with 1% Al expression levels is upregulated with a 5.9-fold change [59]. As an example, TiO_2_ NPs concentrations of 0.1%, 0.5%, 1%, and 2.5% irregularly affected miR156a expression in *Panicum virgatum* L. plants, first increasing and then decreasing [60].

The effect of CuO NPs on the miRNA expression level has not been previously studied. The only difference between the treated and control plants was the presence or absence of Fe_3_O_4_ NPs or CuO NPs, which supports the theory that the changes in plants were caused by this effect of the NPs. Furthermore, Zhu et al. [61] confirmed that 20 nm Fe_3_O_4_ NPs can penetrate into pumpkin cells, translocate, and accumulate in the plant tissues. Moreover, it has been proven that 25 nm Fe_3_O_4_ NPs can penetrate flax callus culture cells [62]. Also, the translocation of 40 nm CuO NPs in the rice roots was observed [63].

For the first time, a study was performed comparing the 17 mg/L, 35 mg/L, and 70 mg/L concentrations of Fe_3_O_4_ and CuO NPs on barley varieties with the *mlo* gene, examining miR156a expression levels. These results suggest that miRNA expression levels in barley varieties treated with different NPs is genotype dependent, as Fe_3_O_4_ NPs at different concentrations only increased the miRNA expression level in the “*KWS Olof*” variety, while CuO NPs at different concentrations increased the miRNA level in the “Marthe” variety.

As copper and iron are often used in agriculture as a nutrient, the presented results could be used in the future to accept new technology for crop plants to increase resistance against fungal pathogens through an increase in miRNA expression levels. Also, nutrition with iron and copper can increase *mlo*-based resistance, which will increase the resistance of plants to powdery mildew. It is possible that in our study, NP increased the resistance to pathogens in plants with *mlo* genes because the increasing miR156a level in barley varieties is established. The role of miR156 in barley has not been fully investigated, but the use of NP is likely to increase the resistance of barley to pathogens. It is necessary to complete further studies with barley varieties with *mlo* genes and without *mlo* genes and to examine different miRNA as well as miR156a expression and their role in plant resistance to infections.

## 4. Conclusion

The results of this study showed that Fe_3_O_4_ and CuO NPs had different effects on plant morphology—Fe_3_O_4_ NPs compared to CuO NPs irregularly increased plant shoot and root lengths and fresh plant biomass. CuO NPs reduced barley seedling shoot and root lengths. The Fe_3_O_4_ and CuO NPs at 17 mg/L, 35 mg/L, and 70 mg/L concentrations mostly insignificantly affected the genome template stability in different varieties of barley. Comparing the effect of Fe_3_O_4_ and CuO NP on barley varieties, CuO NPs increased miRNA levels in plants, which is likely to affect plant resistance to pathogens. To the best of our knowledge, this is the first study aiming to determine the genotoxicity of CuO NPs in plants. Also, no previous studies have been performed to detect changes to the barley genome when treated with Fe_3_O_4_ NPs. The miRNA expression level of barley varieties using NPs at different concentrations is genome dependent. Future studies are necessary to analyse the effect of miR156 and other miRNA expressions in barley mlo varieties and non-mlo varieties seedlings under NP stress, as well as to assess the potential of using NPs for increasing plant resistance against pathogens.

## Figures and Tables

**Table 1:** Results of RAPD analysis; the primers used, number of polymorphic bands in plants treated with 17 mg/L, 35 mg/L, and 70 mg/L of Fe_3_O_4_ and CuO NPs, total number of bands for each primer, and average number of polymorphic bands for every barley variety group.

Primer ID	NPs	Variety	Number of polymorphic bands of 17 mg/L		Number of polymorphic bands of 35 mg/L		Number of polymorphic bands of 70 mg/L		Total number of bands
			a	b	a	b	a	b	
OPA-05	Fe_3_O_4_	“Marthe”	6	0	0	0	0	0	6
		“KWS Olof”	4	0	2	0	5	0	11
	CuO	“Marthe”	14	0	7	0	0	0	21
		“KWS Olof”	0	0	7	0	10	0	17

OPA-07	Fe_3_O_4_	“Marthe”	3	5	0	5	5	5	23
		“KWS Olof”	4	5	6	5	3	1	24
	CuO	“Marthe”	5	5	5	4	3	5	27
		“KWS Olof”	4	3	7	2	5	2	23

OPD-18	Fe_3_O_4_	“Marthe”	5	2	3	2	10	1	23
		“KWS Olof”	3	3	5	4	5	4	24
	CuO	“Marthe”	9	2	4	3	6	2	26
		“KWS Olof”	6	5	4	3	7	5	30
Total polymorphic bands			63	30	50	28	59	25	

Average number of polymorphic bands per experimental group	Fe_3_O_4_	“Marthe”	7		3.33		7		
		“KWS Olof”	6.33		7.33		6		
	CuO	“Marthe”	11.66		7.66		5.33		
		“KWS Olof”	6		7.66		9.66		

A, new bands; b, disappeared bands.

## Data Availability

The data used to support the findings of this study are included within the article.
